# Analysis of the Nonlinear Trends and Non-Stationary Oscillations of Regional Precipitation in Xinjiang, Northwestern China, Using Ensemble Empirical Mode Decomposition

**DOI:** 10.3390/ijerph13030345

**Published:** 2016-03-21

**Authors:** Bin Guo, Zhongsheng Chen, Jinyun Guo, Feng Liu, Chuanfa Chen, Kangli Liu

**Affiliations:** 1 College of Geodesy and Geomatics, Shandong University of Science and Technology, Qingdao 266590, China; luf3286@126.com (F.L.); liukangli111@sina.com (K.L.); 2 State Key Laboratory of Mining Disaster Prevention and Control Co-founded by Shandong Province and Ministry of Science & Technology, Shandong University of Science and Technology, Qingdao 266590, China; 3 State Key Laboratory of Desert and Oasis Ecology, Xinjiang Institute of Ecology and Geography, Chinese Academy of Sciences, Urumqi 830011, China; zihan6688@126.com; 4 Key Laboratory of Surveying and Mapping Technology on Island and Reef, National Administration of Surveying, Mapping and Geoinfomation, Shandong University of Science and Technology, Qingdao 266590, China; 5 Key Laboratory of Geographic Information Science Ministry of Education, East China Normal University, Shanghai 200241, China

**Keywords:** ensemble empirical mode decomposition, precipitation, intrinsic mode function, nonlinear characteristics, Xinjiang

## Abstract

Changes in precipitation could have crucial influences on the regional water resources in arid regions such as Xinjiang. It is necessary to understand the intrinsic multi-scale variations of precipitation in different parts of Xinjiang in the context of climate change. In this study, based on precipitation data from 53 meteorological stations in Xinjiang during 1960–2012, we investigated the intrinsic multi-scale characteristics of precipitation variability using an adaptive method named ensemble empirical mode decomposition (EEMD). Obvious non-linear upward trends in precipitation were found in the north, south, east and the entire Xinjiang. Changes in precipitation in Xinjiang exhibited significant inter-annual scale (quasi-2 and quasi-6 years) and inter-decadal scale (quasi-12 and quasi-23 years). Moreover, the 2–3-year quasi-periodic fluctuation was dominant in regional precipitation and the inter-annual variation had a considerable effect on the regional-scale precipitation variation in Xinjiang. We also found that there were distinctive spatial differences in variation trends and turning points of precipitation in Xinjiang. The results of this study indicated that compared to traditional decomposition methods, the EEMD method, without using any *a priori* determined basis functions, could effectively extract the reliable multi-scale fluctuations and reveal the intrinsic oscillation properties of climate elements.

## 1. Introduction

Climate change is significantly affecting water resources, industrial activities, crop production, natural systems [1] as well as public health [2,3,4], and these effects are particularly intense in arid regions [5,6,7]. The Intergovernmental Panel on Climate Change (IPCC) Fifth Assessment Report (AR5) stated that the global mean surface temperature increased by 0.85 °C during the last 130 years (1880–2012) [8]. Global warming may intensify the hydrological cycle [9,10] and further increase the occurrence of precipitation extremes [11], which in turn exacerbate floods and droughts in many regions [12,13]. Moreover, precipitation changes, especially intense precipitation or droughts have a profound impact on public health [2,3,4]. For example, Curriero *et al.* [2] revealed that heavy precipitation events contributed significantly to the risk of waterborne disease outbreaks. Cheng *et al.* [3] concluded that extreme precipitation had greater and longer-lasting effects on hand, foot and mouth disease (HFMD) in children aged 0–4 years, female children and urban children.

Located in the hinterland of the Eurasia continent, Xinjiang is sensitive to global climate change [14]. Generally, Xinjiang is divided into three distinct sub-regions (*i.e.*, northern, southern and eastern Xinjiang) according to its natural settings and climate patterns. Northern Xinjiang is semiarid and has the lowest temperature and the largest amount of precipitation, while southern and eastern Xinjiang are characterized by a typical continental arid climate and have higher temperature and less precipitation [14]. With global warming, the regional temperature in Xinjiang has been increasing during the past several decades [6,15], accompanied by an increase in annual precipitation as well [16,17]. The regional climate has shifted from warm-dry to warm-wet since the mid-1980s [6]. In addition, floods of melt-water from glacier and snow cover occur more frequently in northern Xinjiang, while the occurrence of droughts is increasing in the arid southern and eastern regions of Xinjiang [17]. As one of the most important irrigated agriculture regions in China, Xinjiang is suffering from serious water shortages and constitutes a vulnerable ecosystem [17]. Therefore, the changes in precipitation could have crucial influences on the regional ecological environments and sustainable socioeconomic development. Hence, understanding the variations of precipitation in different parts of Xinjiang in the context of climate change is of great importance for the regional ecology, economy and agriculture.

It is generally accepted that climate system is a highly complex nonlinear system [18,19,20,21], and most of the long-term variations in climatic factors, such as temperature and precipitation, exhibit nonlinear and non-stationary complex processes, accompanied by various periodic oscillations or time scales [19,22,23,24]. Analysis of such time varying climate signals is not an easy process which requires decomposition of the process into individual components and to analyze each component separately [25]. In the past twenty years, a large number of studies have been conducted to examine the variability and trends in global and regional climate over the past several decades [11,12,15,26,27,28,29]. However, many studies focused on averaged trends over that time span using conventional statistical methods, such as straight line fitting, which can extract the sign and rate of climate change trends in the past [15,28,30]. Moreover, traditional decomposition methods, including the Fourier transform (FT) and Wavelet analysis (WA), employ basis functions determined *a priori*, which may accurately reflect the characteristics of a time series in some segments but not in other segments of a non-stationary time series [22,25,29]. The shortcomings of these conventional means are that, in theory, the applied time series is assumed to be stationary [21]. Generally, there is still a lack of effective methods available to reveal the change of climate system in its intrinsic form.

With the rapid development of advanced signal decomposition techniques, ensemble empirical mode decomposition (EEMD) has been presented by Wu and Huang to reveal any hidden intrinsic non-stationary oscillation structures in a time series [31]. Contrary to most of the previous decomposition methods (such as FT and WA), EEMD emphasizes the self-adaptability and temporal locality for describing nonlinear and non-stationary time series data, without using any *a priori* determined basis functions [31,32]. As a substantial improvement of empirical mode decomposition (EMD), EEMD can effectively eliminate the mode (or scale) mixing problem of EMD and also be able to separate the intrinsic mode function (IMF) components on different time scales and trend component from original time series [31]. The IMF components represent the intrinsic oscillatory mode embedded in the signal and work as the basis functions, which are determined by the signal itself, rather than predetermined kernels [31]. The trend component which is obtained by using the EEMD represents the variation trend of the original time series. Compared to other conventional methods, the EEMD method can more efficiently extract periodic information and trends [28,33]. Recently, some significant achievements have been obtained by using this method in hydrological research [22,33,34,35] and climate change studies [18,19,29,36,37,38]. Consequently, compared to previous studies [14,15,17], it is critical to reveal the multi-scale variation characteristics of precipitation in terms of natural oscillatory patterns and trends using the EEMD method in the study area.

The primary objectives of this study were: (1) to detect the multi-scale evolution of regional precipitation variation in Xinjiang during the last 50 years; (2) to explore the contributions of oscillations on different time scales to the overall precipitation variation and the spatial differences in variation and turning points of precipitation. The results of the current study will provide a baseline for sustainable water management in relation to the changing environment in Xinjiang.

## 2. Materials and Methods

### 2.1. Study Area Description

**Figure 1:**
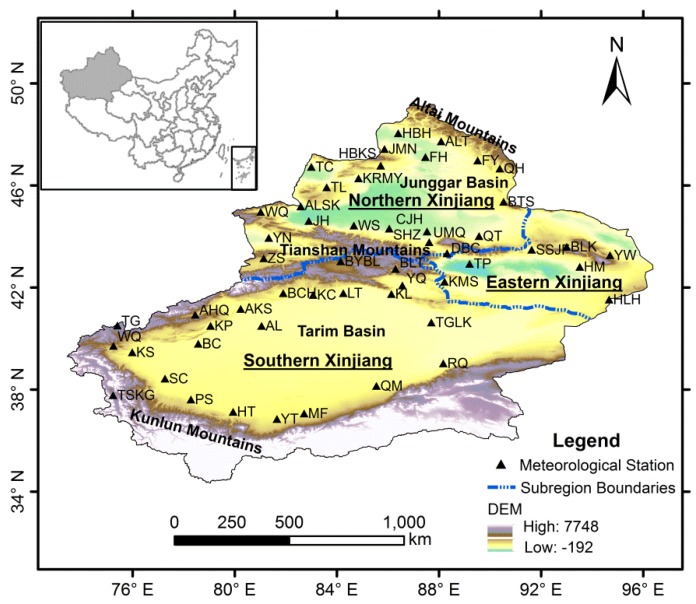
A map of Xinjiang and locations of meteorological stations.

Xinjiang Province, consisting of northern, southern and eastern parts, is located in northwestern China. It lies approximately between 34°25′ N and 49°10′ N latitude and between 73°40′ E and 96°23′ E longitude (Figure 1). With an area of 1,660,400 km^2^, Xinjiang is the largest provincial level administrative region in China, occupying one-sixth of the total land area. There are three mountain ranges from north to south in Xinjiang: the Altai Mountains, the Tianshan Mountains and the Kunlun Mountains. Two major basins (the northern Junggar Basin and southern Tarim Basin) lie among these three mountain ranges [6]. Located in the middle part of Tarim Basin, Taklamakan Desert is the largest desert in China and the second largest shifting sand desert in the world. Gurbantunggut Desert, the second largest desert in China, lies in the central section of Junggar Basin. The Tianshan Mountains in the middle divide Xinjiang Province into two parts, named the northern and southern Xinjiang [15]. Northern Xinjiang covers Altay, Tacheng, Ili, Bortala Mongolian Autonomous Prefecture, Urumqi and Changji. Southern Xinjiang includes Aksu, Kashgar, Hotan, Bayingolin Mongolian Autonomous Prefecture and Kizilsu Kirgiz Autonomous Prefecture. Turpan-Hami Basin lies in the east part of Xinjiang, named the eastern Xinjiang. The climate in Xinjiang is featured by continental arid conditions. The total annual precipitation in Xinjiang is about 134 mm and is distributed unevenly which decreases from northwest to southeast, from windward slopes to leeward slopes and from the mountain areas to the basins. The total annual precipitations in northern, southern and eastern Xinjiang are 197 mm, 94 mm and 72 mm, respectively, with annual average temperatures of these three parts range from 4 °C to 8 °C, 10 °C to 13 °C [39] and is 5 °C to 15 °C, respectively. The average temperature in July is over 33 °C in Turpan, and the absolute maximum temperature is 49.6 °C, making Turpan the hottest place in China. The evaporation in Xinjiang is very strong (with the mean annual pan evaporation between 1000 and 4500 mm) and is 500–1000 mm higher than other regions of the same latitude in China [15].

### 2.2. Data Description

In this study, the annual precipitation data of 53 meteorological stations in Xinjiang during the period of 1961–2012 were collected from China Meteorological Data Sharing Service System [40]. All these meteorological stations selected for the study had the most completed dataset with acceptable data quality control. The strict data quality control and homogeneity assessment were performed using software RHtestV3, which can be used to detect, and adjust for, multiple shifts that could exist in a data series that may be associated with systematic errors (e.g., resulting from instrument replacement, migration of stations and operating errors of the observer) [41]. The locations of these meteorological stations in Xinjiang are shown in Figure 1, and their longitudes, latitudes and altitudes are listed in Table 1.

### 2.3. Ensemble Empirical Mode Decomposition (EEMD)

#### 2.3.1. EEMD Algorithm

The EMD method is an efficient and adaptive tool for decomposing nonlinear and non-stationary signals into a series of intrinsic mode function components (IMFs) through the sifting process [32]. In the EMD approach, the highest frequency oscillation is identified as the first IMF; the frequencies of the following IMFs decrease on each step [42]. Generally the IMF component with the lowest frequency (RES) represents the general trend or mean of the original signal. For a given data *x*(*t*), the EMD procedure is as follows [32]:

First, all local maxima and minima of *x*(*t*) must be identified. Then, all local maxima are connected by a cubic spline to form the upper envelope. The procedure is repeated for the local minima to produce the lower envelope [32]. The mean value of the upper and the lower envelopes, *m*_1_(*t*), is subtracted from the original time series, *x*(*t*), and denoted as *h*_1_(*t*), *i.e.*,:

(1) $$h_{1}(t)=x(t)-m_{1}(t)$$

where, *h*_1_(*t*) could be the first IMF. Each IMF should satisfy the following two conditions: (1) in the whole data set, the number of extrema must be equal to the number of zero-crossings or differ from it at most by one; and (2) at any point, the mean value of the two envelopes determined by the local maxima and minima must be zero [32]. However, if *h*_1_(*t*) does not satisfy the IMF conditions, the sifting process must be repeated. In subsequent processes, *h*_1_(*t*) is treated as the new data, then:

(2) $$h_{11}(t)=h_{1}(t)-m_{11}(t)$$

where *m*_11_(*t*) is the mean of the envelopes with *h*_1_(*t*) replacing the data *x*(*t*) in the first iteration. This process can be repeated up to *k* times:

(3) $$h_{1k}(t)=h_{1(k-1)}(t)-m_{1}{}_{k}(t)$$

The IMF will become a constant-amplitude frequency-modulated function if the sifting process is repeated too many times, possibly rendering the results physically less meaningful. Consequently, a criterion for the sifting process to stop should be established when it is guaranteed that the IMF retains enough physical sense of both amplitude and frequency modulations [32,43]. This can be accomplished by limiting the size of the standard deviation, *SD*, computed from the two consecutive sifting results as [32]:

(4) $$SD=\sum_{t=0}^{T}\left[\frac{|(h_{1\left(k-1\right)}(t)-h_{1k}(t)){|}^{2}}{h^{2}{}_{1\left(k-1\right)}(t)}\right]$$

A typical value for *SD* can be set between 0.2 and 0.3 [32]. In the present study, we decomposed the data using EEMD with different *SD* values (*i.e.*, 0.2 and 0.3) but found few differences in the results. In order to facilitate the analysis, we only displayed the results of *SD* = 0.2. The first IMF is obtained when *SD* is smaller than the defined threshold:

(5) $$c_{1}(t)=h_{1k}(t)$$

Overall, *c*_1_(*t*) should contain the finest scale or the shortest period component of the data set. And the residue *r*_1_(*t*) is calculated as:

(6) $$r_{1}(t)=x(t)-c_{1}(t)$$

The residue *r*_1_(*t*) is treated as the signal to be decomposed and the above steps are repeated to obtain *c*_2_(*t*), *c*_3_(*t*), …, *c_n_*(*t*). The procedure stops when the residue *r_n_*(*t*) becomes a monotonic function from which no more IMF can be extracted [32].

After the sifting processing, the original data set can be described as the sum of the IMFs and the final residue:

(7) $$x(t)=\sum_{i=1}^{n}c_{i}(t)+r_{n}(t)$$

where *n* is the number of IMFs and it is close to Log_2_*N*-1, with *N* being the number of total data points [42]. *c_i_*(*t*) is the *i*th IMF and *r_n_*(*t*) is the final residue (*i.e.*, trend component).

However, one potential shortcoming of EMD is mode mixing. Mode mixing not only causes serious aliasing in the time-frequency distribution but also causes the physical meaning of individual IMFs to be unclear. To avoid mode mixing problem in EMD, a new noise assisted data analysis method named ensemble empirical mode decomposition (EEMD), has been proposed [31]. The EEMD procedure is as follows: (1) add white noises of finite amplitude to the signal; (2) decompose the data plus the white noise into IMFs using EMD; (3) repeat the above steps with different sets of white noise each time; (4) find the mean of each IMF obtained in different trials to arrive at the final IMFs.

One of the crucial issues in the EEMD method to prevent mode mixing is the amplitude of the noise added. The scaling factor should be neither too small nor too large. It has been reported that the optimum amplitude of the noise to be added is in the range of 0.1–0.4 time the standard deviation of the original signal [31]. In addition, the ensemble size should increase when the amplitude of noise increases so as to reduce the contribution of added noise in the decomposed results [31]. We examined the sensitivities of these parameters (*i.e.*, the amplitude of the added noise and the number of ensemble realizations) on the decomposition results based on precipitation time series for Xinjiang during 1960–2012. Our experiments results showed that the EEMD results are insensitive to these parameter choices (e.g., using 1500 ensemble realizations does not change the results; see [31] for more details). In this present study, we use 1000 ensemble realizations with the amplitude of the added noise of 0.3 time standard deviation of the original signal.

#### 2.3.2. Significance Test of IMF Components

To see whether an IMF for EEMD contains a true signal or just a random noise component, the Monte-Carlo method was used to perform a significance test [44,45]. The statistical significance test for IMF components derived from white noise was established by Wu and Huang [44,45]. The true signals were determined by examining the more detailed distribution of the energy with respect to the period in the form of spectral function. The energy density of the *i*th IMF component (*E_i_*) can be defined as follows [31]:

(8) $$E_{i}=\frac{1}{N}{\sum_{t=1}^{N}\left|c_{i}(t)\right|}^{2}$$

where *N* is the length of the IMF component and *c_i_*(*t*) denotes the *i*th IMF component.

There exists a simple equation that relates the energy density and the mean oscillation period (*T_i_*) [31]:

(9) $$\ln E_{i}+\ln T_{i}=0$$

If the IMF energy of the climate signal with a certain mean period exceeds the upper limit of a certain confidence interval, we assume that the corresponding IMF contains statistically significant information at that selected confidence level (e.g., 90%). The results of the EEMD of the precipitation data in Xinjiang were tested employing the suggested approach to determine whether their IMFs contained possible physical meanings [42,45] at the 90% and 95% confidence levels.

### 2.4. The Mann-Kendall (M-K) Test

Climatic and hydrologic time series possess some of the following characteristics: no normal data, missing values, censoring, and serial dependence [46]. Parametric statistical tests for detecting trends are commonly confounded [46]. However, the M-K nonparametric test, suggested by the World Meteorological Organization, has been widely used for determining the occurrence of abrupt turning points of meteorological and hydrological series [47,48]. This method can simply confirm the starting time of abrupt changes and identify the area of abrupt changes [49,50]. In this current study, we apply M-K nonparametric test to determine the abrupt turning points of precipitation series [49,50].

Let *x*_1_, ..., *x_N_* be the data points. For each element *x_I_*, the numbers *W_I_* of elements *x_J_* preceding it (*J* < *I*) such that *x_J_* < *x_I_* are computed. Under the null hypothesis (no abrupt turning point), the normally distributed statistic *S_l_* can be defined as:

(10) $$S_{l}=\sum_{I=1}^{l}W_{I},2\leq l\leq N$$

Mean and variance of the normally distributed statistic *S_l_* can be given by the following formulas:

(11) $$\bar{S_{l}}=E(S_{l})=l(l-1)/4$$

(12) $$var(S_{l})=l(l-1)(2l+5)/72$$

The normalized variable statistic *UF_l_* is estimated as follows:

(13) $$UF_{l}=(S_{l}-\bar{S_{l}})/\sqrt{\mathrm{var}(S_{l})}$$

The normalized variable statistic *UF_l_* is the forward sequence, and the backward sequence *UB_l_* is calculated using the same equation but with a reversed series of data. When the null hypothesis is rejected (*i.e.*, if any of the points in the forward sequence is outside the confidence interval), the detection of an increasing (*UF_l_* > 0) or a decreasing (*UF_l_* < 0) trend is indicated. The sequential version of the test used here enables detection of the approximate time of occurrence of the trend change by locating the intersection of the forward and backward curves of the test statistic. If the intersection occurs within the confidence interval, then it indicates an abrupt turning point.

### 2.5. Variance Contribution Rate (VCR)

The variance contribution rate (*VCR*) illustrates the effects of the frequency of the fluctuation and amplitude at different scales on the overall characteristics of the original signal [24,36]. The variance contribution rate of the *i*th IMF component (*VCR_i_*) can be calculated via the following formula:

(14) $$VCR_{i}=\frac{\mathrm{var}(c_{i}(t))}{\sum_{i=1}^{n}\mathrm{var}(c_{i}(t))+\mathrm{var}(r_{n}(t))}\times 100\%$$

where var(*c_i_*(*t*)) and var(*r_n_*(*t*)) represent the variances of the *i*th IMF component and the final residue, respectively.

## 3. Results and Discussion

### 3.1. Inter-Annual Variation of Precipitation

**Figure 2:**
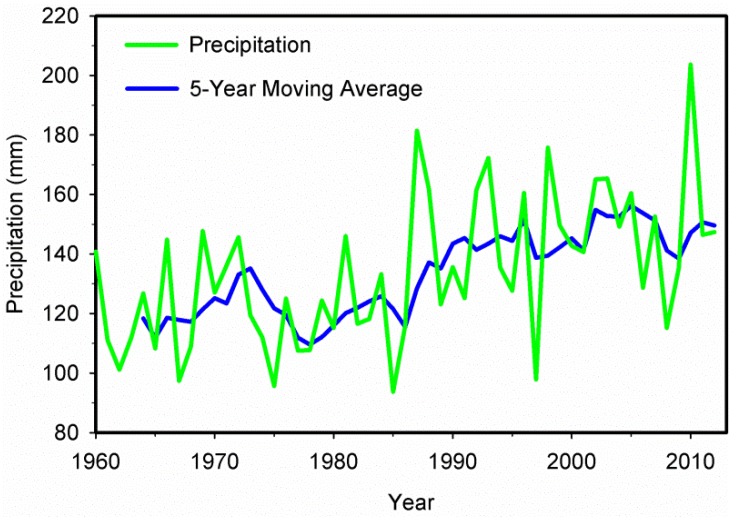
The precipitation time series during 1960–2012 in Xinjiang.

**Figure 3:**
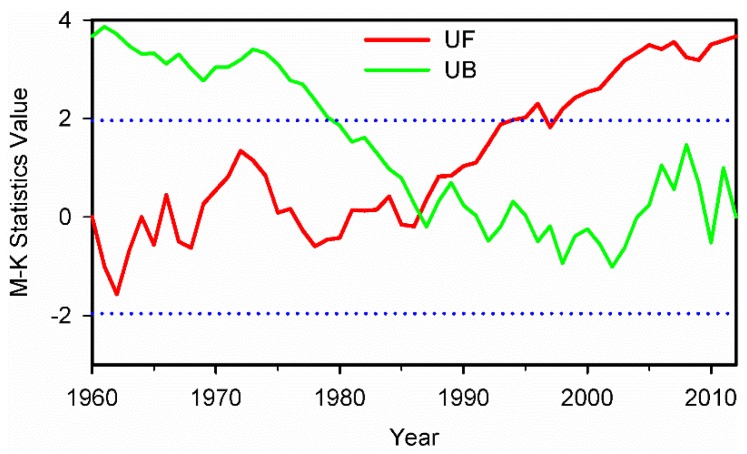
The M-K analysis result of average annual precipitation at 95% confidence level (the blue dash dotted lines) during 1960–2012 in Xinjiang.

The precipitation time series for Xinjiang during 1960–2012 is shown in Figure 2. From 1960 to 2012, the precipitation in this region presented an overall increasing trend with fluctuations. Figure 3 shows the M-K analysis of annual precipitation at 95% confidence level in Xinjiang. The intersection of the curves indicates that the year of 1987 was the turning point when the precipitation transitioned from relatively dry to wet, which is coherent with the result of Shi *et al.* [6]. The average annual precipitation increased from 119.9 mm during 1960–1986 to 148.5 mm during 1987–2012. During 1960–1986, the precipitation was lower and presented a gradual increasing trend. Afterwards, it became obviously higher during the 1990s, with the largest differences up to 78 mm and more extreme precipitation events occurred. The overall amount of annual precipitation during the first ten years of the 21st century remained at a relatively high level while the extreme precipitation events continuously increased. The increase of atmospheric water vapor content and the accelerating water cycle are the possible causes of an increase in precipitation in Xinjiang [6].

### 3.2. Multi-Scale Temporal Variation of Precipitation

**Figure 4:**
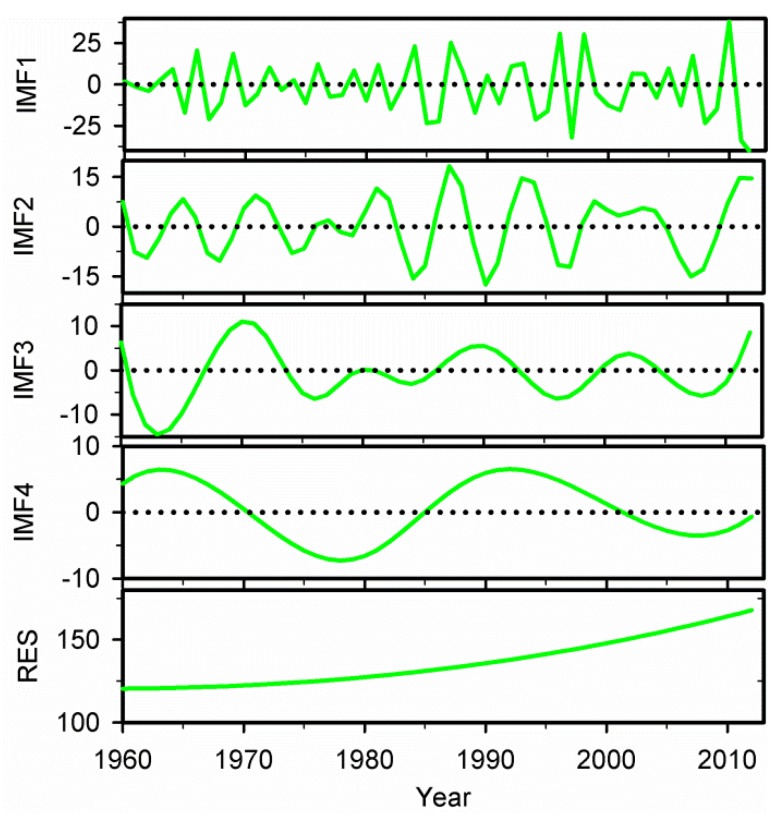
The IMFs and trend component of average annual precipitation time series in Xinjiang during 1960–2012 by EEMD.

**Figure 5:**
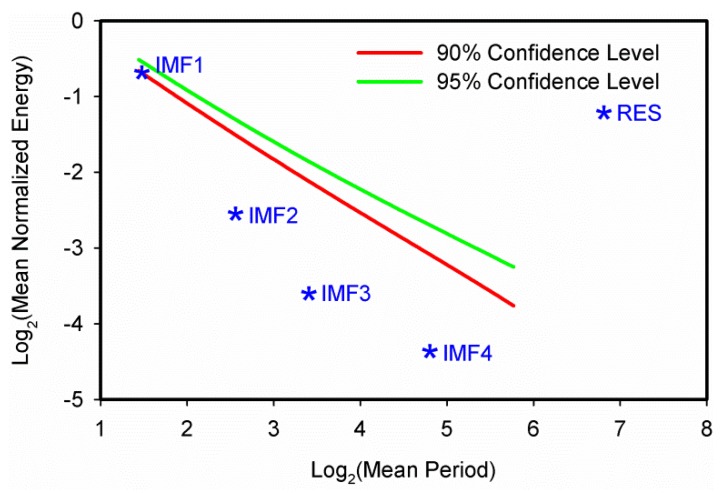
Significant test results for the IMFs of average annual precipitation time series during 1960–2012 in Xinjiang.

The annual precipitation series in Xinjiang during 1960–2012 were decomposed into four IMFs and one trend component using the EEMD method (Figure 4). Each IMF reflects the characteristics of fluctuation in different time scales from high frequency to low frequency. The first IMF (IMF1) contains the highest frequency or the finest time scale and that time scale increases as the index *j* of IMF*j* increases. And the component with the lowest frequency generally represents the overall trend of the original signal over time. In general, each IMF has its own physical meaning, which reflects the inherent oscillation in the original signal [32]. The inherently different time scales can be determined by the Monte Carlo method mentioned above, and the confidence level indicates the strength of inherently different time scales [45]. As shown in Figure 4 and Figure 5, the precipitation changes in Xinjiang presented relatively stable quasi-periodic oscillation from 1960 to 2012. During the study period, the regional precipitation variation had the strongest 2-year (IMF1) and weak 6-year (IMF2) quasi-periodic fluctuations at the inter-annual scale but unobvious 12-year (IMF3) and 23-year (IMF4) quasi-periodic fluctuations at the multi-decadal scale.

Figure 5 shows the results of the significance test of all the extracted IMFs which reveals that the first and trend components are highly significant compared to other components. It was found that IMF1, which represented the quasi-2 years variability of the original signal, fell between the 90% and 95% confidence level, implying that IMF1 was statistically significant at the 90% confidence level and contained the most information with actual physical meaning. However, IMF2, IMF3 and IMF4, which represented the 6-year, 12-year and 23-year quasi-periodic oscillations of the data, respectively, fell below the 90% confidence level, suggesting that they all contained less information with actual physical meaning.

These multi-scale oscillations of precipitation reflect not only the periodic variations of the climate system under external forcing but also the non-linear feedback of the climate system [24,36]. The tropospheric biennial oscillation (TBO) with roughly a 2–3 years cycle is the basic characteristic of inter-annual variation of atmospheric circulation [51]. A previous study showed that a significantly 2–3 years cycle in the annual precipitation change in the mid-latitude Asia may be related to the TBO [51]. In our study, a significant TBO of the precipitation variation in Xinjiang was also confirmed. The 6-year quasi-periodic fluctuation is related to the El Niño Southern Oscillation (ENSO) and North Atlantic Oscillation (NAO) [52,53,54], indicating that the ENSO and NAO events have an obvious impact on the precipitation variation in Xinjiang. The 12-year and 23-year quasi-periodic fluctuations are associated with solar activity, such as the well-known quasi-11-year period and the quasi-22-year magnetic period of the sunspot numbers [55,56]. More detailed relationships between multi-scale oscillations of precipitation and large-scale oscillations will be examined in our future study.

Dai *et al.* analyzed the multi-scale features of precipitation in Xinjiang from 12 stations during 1951–2005 by wavelet decomposition and detected obvious cyclic periods of 2 and 6 years at the inter-annual scale and weaker cyclic period of 11 and 16 years at the inter-decadal scale in the series of precipitation [57]. With respect to the inter-annual scale characteristics, the result reported by Dai *et al.* [57] is consistent with our study. However, there are some differences on the inter-decadal scale. The pronounced discrepancies may be attributed to the different selection of stations and study periods. To verify the discrepancies of the decomposition results from these two methods, wavelet decomposition was used to detect the multi-scale features of precipitation from the 53 meteorological stations in Xinjiang during 1960–2012. We selected different wavelet bases and decomposition levels for the decomposition of precipitation in Xinjiang. It was found that the decomposition results were remarkably different if various wavelet bases and decomposition levels were selected (not presented herein), indicating that the wavelet transform is not adaptive [36]. The wavelet transform results based on Morlet wavelet showed that there were obvious cyclic periods of 3 and 7 years at the inter-annual scale and weak cyclic period of 13 and 25 years at the inter-decadal scale in the series of precipitation. Therefore, the differences between the result reported by Dai *et al.* [57] and our study can be attributed to not only the selection of stations and study periods but also the methods themselves. Compared to wavelet transform, EEMD is empirical, intuitive, direct and adaptive, without using any predetermined basis functions, but only based on the principle of local scale separation [22,32,33].

### 3.3. The Variance Contribution Rate of IMFs and Trend Component

The effect of the frequency of the fluctuation and amplitude at different scales on the overall characteristics of the original signal can be represented by the variance contribution rate [36]. It should be noted that all IMFs and the trend component are taken in the calculations of the variance contribution rate to maintain the total energy of the original signal.

The variance contribution rate of each IMF and trend component of the precipitation anomaly is presented in Table 2. The variance contribution rate of IMF1 ranked first among all the IMFs, accounting for more than 47.00% of the total variability. And the amplitude of the regional precipitation oscillated strongly from a decrease-increase-decrease-increase trend which was obviously higher during the late-1980s, 1990s and the early-2010s than that of other periods (Figure 4). According to the result of variance contribution rate, IMF2 contributed approximately 12.59% to the total precipitation variability. And a relatively larger precipitation amplitude during the late-1980s and the early-1990s was examined on this time scale (Figure 4). The variance contribution rate of IMF3 was 5.15% of the original signal. A relatively smaller amplitude of precipitation was found after the early-1970s on this time scale (Figure 4). However, the variance contribution rate of IMF4 was only 3.13%, which contributed less to the total precipitation variability. At the same time, the variance contribution rate of the trend component accounted for more than 32.13% of the original signal, which implies that the overall trend component also contained much more of the variability. Huang *et al.* stated that the residue was often treated as the deterministic long-term behavior [32]. In addition to inter-annual and multi-decadal variability, there was an overall nonlinear upward trend in annual precipitation in Xinjiang during 1960–2012. The previous studies have shown that precipitation in other regions have also experienced complex and nonlinear processes [11,24,58,59], indicating that the nonlinear process for precipitation is a global issue.

**Figure 6:**
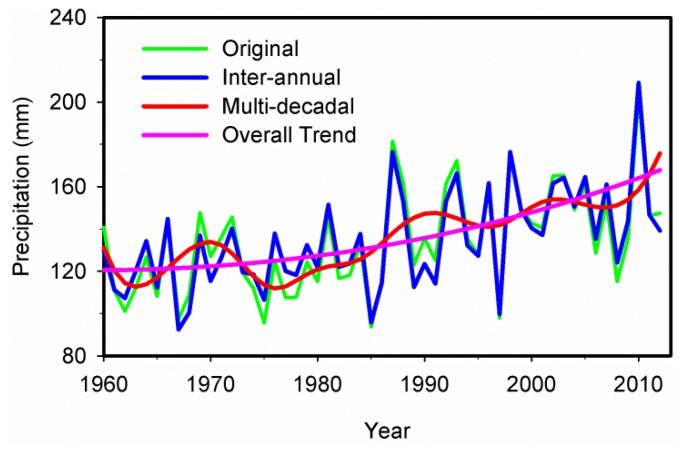
The average annual precipitation time series and its inter-annual trend, multi-decadal trend and overall adaptive trend in Xinjiang.

Table 2 also shows that the inter-annual oscillations are more dominant than multi-decadal oscillations in regional precipitation variation. Various trends, including the inter-annual trend (the sum of IMF1, IMF2 and the trend component), the multi-decadal trend (the sum of IMF3, IMF4 and the trend component), and the overall adaptive trend, were plotted in Figure 6. The inter-annual trend is obtained by IMF1 and IMF2 plus trend component, while multi-decadal trend is obtained by IMF3 and IMF4 plus trend component. All the different trends in comparison with the original precipitation time series were illustrated in Figure 6. It was obvious that the reconstructed inter-annual variation trend of precipitation was almost consistent with the fluctuations of the original precipitation anomaly series during the study period. However, variability with respect to the multi-decadal trend (*i.e.*, the sum of IMF3, IMF4 and the trend component) cannot capture the anomaly series of precipitation perfectly, which may be attributed to the exclusion of small-scale fluctuations from the reconstructed multi-decadal precipitation variation. In addition, the variability with respect to the intrinsically determined overall trend (*i.e.*, trend component derived by the EEMD) can fully reflect the regional variation in precipitation in Xinjiang during 1960–2012, indicating a major improvement over the linear trend [42,45].

### 3.4. Spatial Distribution of Variation of Precipitation

**Figure 7:**
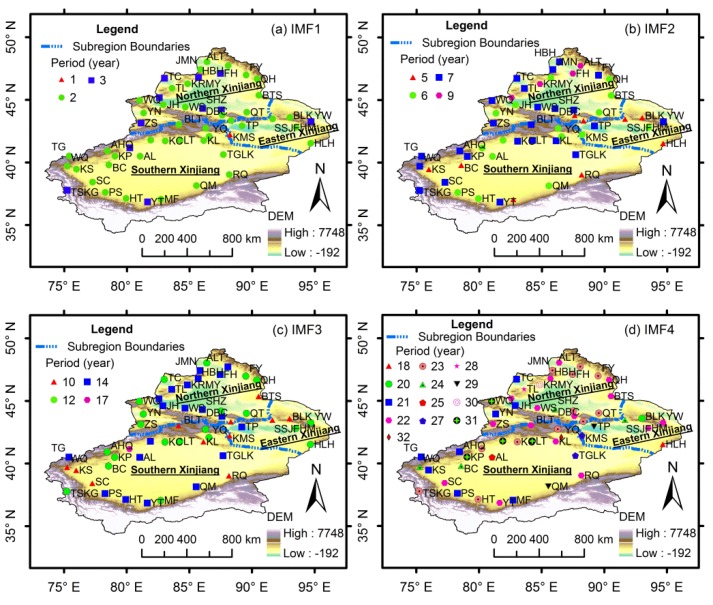
Spatial distribution of quasi-periodic fluctuations of (**a**) IMF1; (**b**) IMF2; (**c**) IMF3 and (**d**) IMF4 in Xinjiang.

The annual precipitation series for each meteorological station in Xinjiang during 1960–2012 were also decomposed into four IMFs and one trend component using the EEMD method. The precipitation changes for each station also presented relatively stable quasi-periodic oscillation from 1960 to 2012. Figure 7 shows the spatial distribution of quasi-periodic fluctuations at the inter-annual scale (*i.e.*, IMF1 and IMF2) and the multi-decadal scale (*i.e.*, IMF3 and IMF4). The annual precipitation in most stations had 2–3-year (IMF1) and 6–7-year (IMF2) quasi-periodic fluctuations at the inter-annual scale (Figure 7a,b). The meteorological stations with 5-year and 9-year quasi-periodic fluctuations (IMF2) were mainly located in the eastern and northwestern Xinjiang, respectively. In addition, the precipitation in most stations had 10–14-year (IMF3) and 21–23-year (IMF4) quasi-periodic fluctuations at the multi-decadal scale (Figure 7c,d).

**Figure 8:**
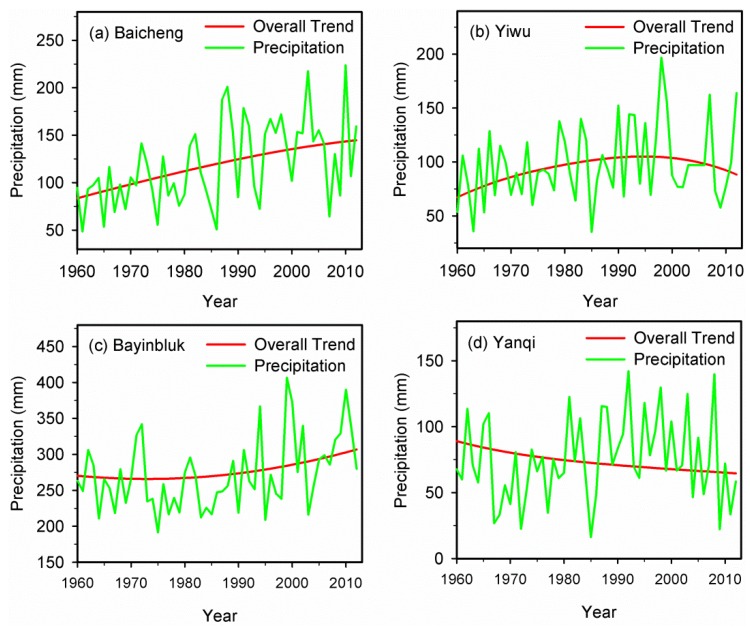
Types of precipitation variation trends in selected typical meteorological stations. (**a**) Baicheng; (**b**) Yiwu; (**c**) Bayinbluk; (**d**) Yanqi.

As mentioned above, the precipitation in Xinjiang showed an overall upward trend during 1960–2012. However, the variation trends differed in sub-regions as a result of the complex topography, circulation types and strength, and other factors. In order to analyze the variation trends of precipitation in each meteorological station in detail, a morphological analysis of the variation trends of precipitation with the method of EEMD has been performed in this study. The result indicated that cases have been found among the 53 stations for each of the four *a priori* conceivable morphological types: the type of increasing, the type of increasing-decreasing, the type of decreasing-increasing and the type of decreasing. The selected four typical meteorological stations are shown in Figure 8. The meteorological stations of Baicheng, Yiwu, Bayinbluk and Yanqi represented the increasing type, the increasing-decreasing type, the decreasing-increasing type and the decreasing type, respectively.

**Figure 9:**
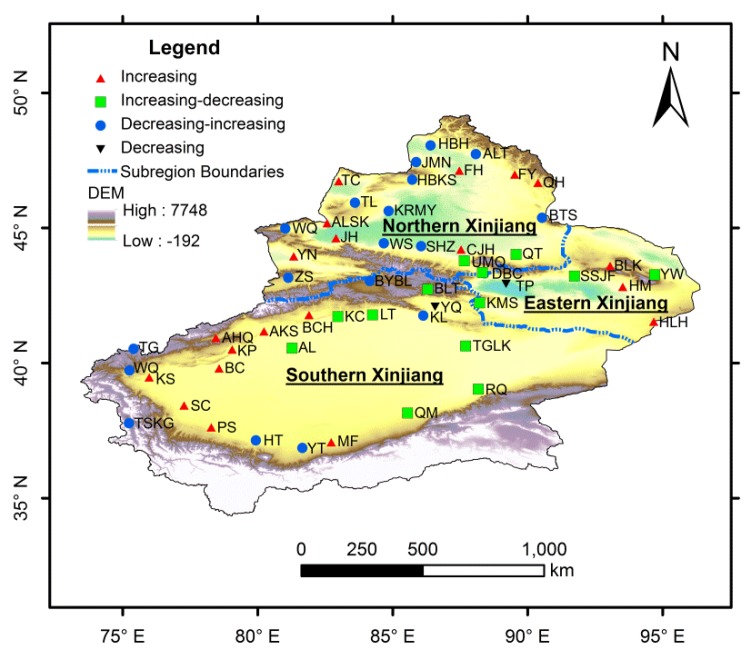
Spatial distribution of the types of variation trends in precipitation in Xinjiang.

Spatial distribution of the types of variation trends in precipitation in Xinjiang is shown in Figure 9. Among all the 53 meteorological stations, there were 20 meteorological stations with the type of increasing, 13 with the increasing-decreasing type, 18 with the decreasing-increasing type and only two with the decreasing type. The meteorological stations with increasing trends were mainly located in the western Xinjiang, such as the southern part of the Ili River Valley and the southern part of the Altay Mountains which were directly related to the westerly circulation, and the Kumul Basin which was likely affected by the Siberian High and westerly circulation. Therefore, the variations of circulation factors were probably a vital reason for the increasing in precipitation in these regions. Stations with increasing-decreasing trend were mainly located in the northern and eastern part of the Tarim Basin and the middle part of Tianshan Mountains, while stations with decreasing-increasing trend were mainly located in the northwestern and southwestern Xinjiang and the south foothills of the central Tianshan Mountains. These two types of variations in precipitation may be mainly influenced by the terrain. However, the precipitation at the stations of Yanqi and Turpan showed an obviously decreasing trend.

**Figure 10:**
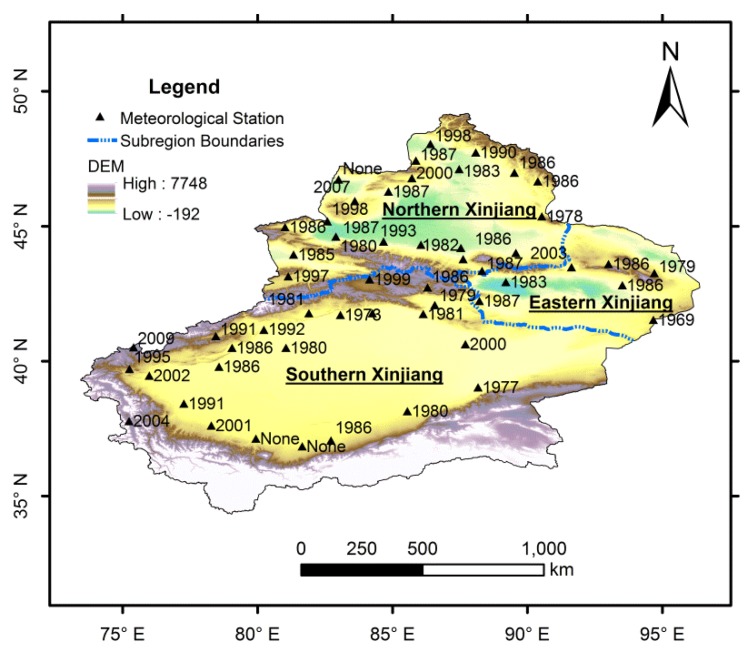
Spatial distribution of the turning points of annual precipitation in Xinjiang.

Figure 10 shows the spatial distribution of the turning points of annual precipitation in Xinjiang. We found that both the variation trends and the turning points differed among all the meteorological stations, indicating that the precipitation variations were not completely synchronized in the entire study area. The overall transition of precipitation in Xinjiang in 1987 was probably attributed to the superimposed effect generated by the variations of precipitation of each station. Generally, the precipitation changes of each station were determined by the inherent mechanism of the regional climate system and the local environment, such as the complex topography, circulation type and strength and human activities. Further studies need to be performed to explore the reasons for the spatial variation of precipitation and detailed interpretation will be conducted in our further publications.

### 3.5. Variation Characteristics of Precipitation in Different Regions of Xinjiang

**Figure 11:**
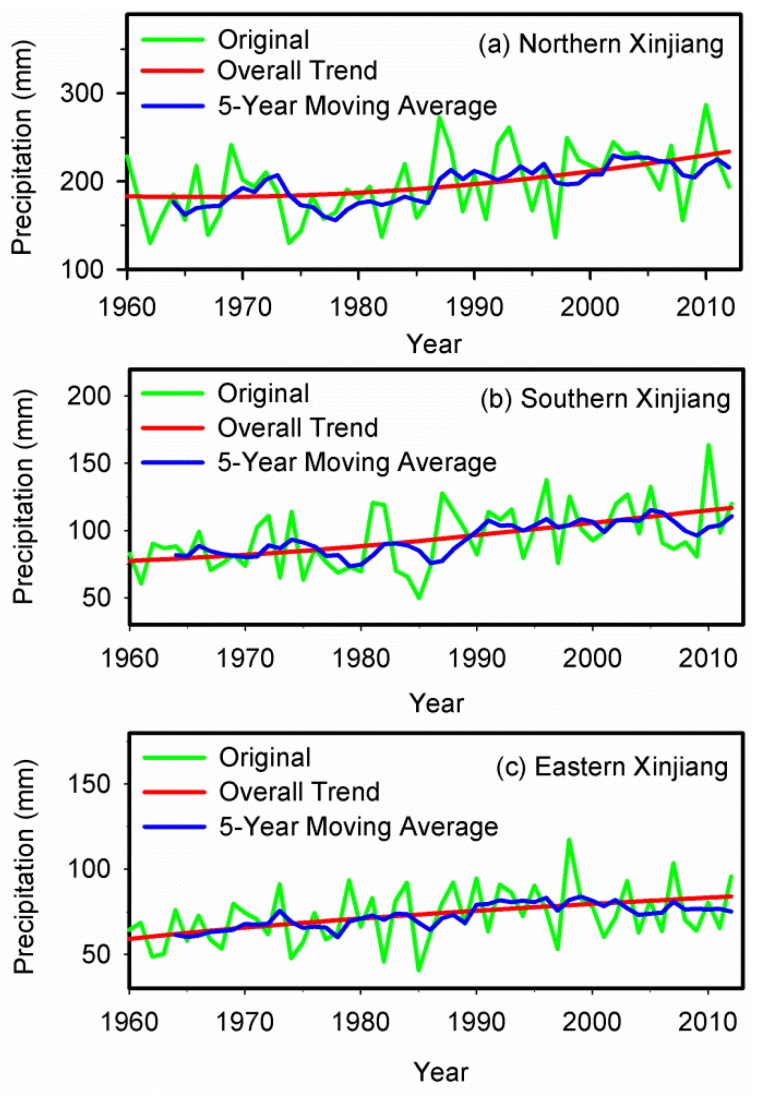
The original average annual precipitation time series and overall trends in (**a**) northern; (**b**) southern and (**c**) eastern Xinjiang during 1960–2012.

The variation characteristics of precipitation in the north, south and east parts of Xinjiang were also analyzed. The annual precipitation series in these three regions during 1960–2012 were all decomposed into four IMFs and one trend component using the EEMD method. The notable non-linear upward trends of regional precipitation were also detected in these three regions (Figure 11). The northern Xinjiang had a rising trend with a mean rate of 10.63 mm per decade, while the precipitation in southern and eastern Xinjiang increased slightly with only 6.90 mm and 3.71 mm per decade, respectively.

The variance contribution rate of each IMF and trend component of the precipitation anomaly in different regions of Xinjiang was also calculated (Table 3). As shown in Table 3, in northern Xinjiang, inter-annual variations of precipitation were detected in the first and second IMFs, which represented 2-year and 7-year quasi-periodic fluctuations, respectively. In addition, inter-decadal variations with 14-year and 25-year periods were detected in the third and fourth IMFs, respectively. The variance contribution rate of IMF1 accounted for 35.09% of the total variability of the precipitation in northern Xinjiang. The significance test results showed that the first and trend components were statistically significant at the 90% confidence level. In southern Xinjiang, there were also the inter-annual (2-year and 5-year quasi-periodic fluctuations) and multi-decadal (10-year and 23-year quasi-periodic fluctuations) variations in precipitation. The variance contribution rate of IMF1 ranked the first of all the IMFs, accounting for 40.47% of the total variability of precipitation. In eastern Xinjiang, the variance contribution rate of IMF1 was 47.93%, followed by the trend component, IMF2, IMF4 and IMF3, which represented the overall trend, 6-year, 21-year and 12-year quasi-periodic fluctuations, respectively. In northern Xinjiang, the first and trend components were also statistically significant at the 90% confidence level in the south and east of Xinjiang.

**Figure 12:**
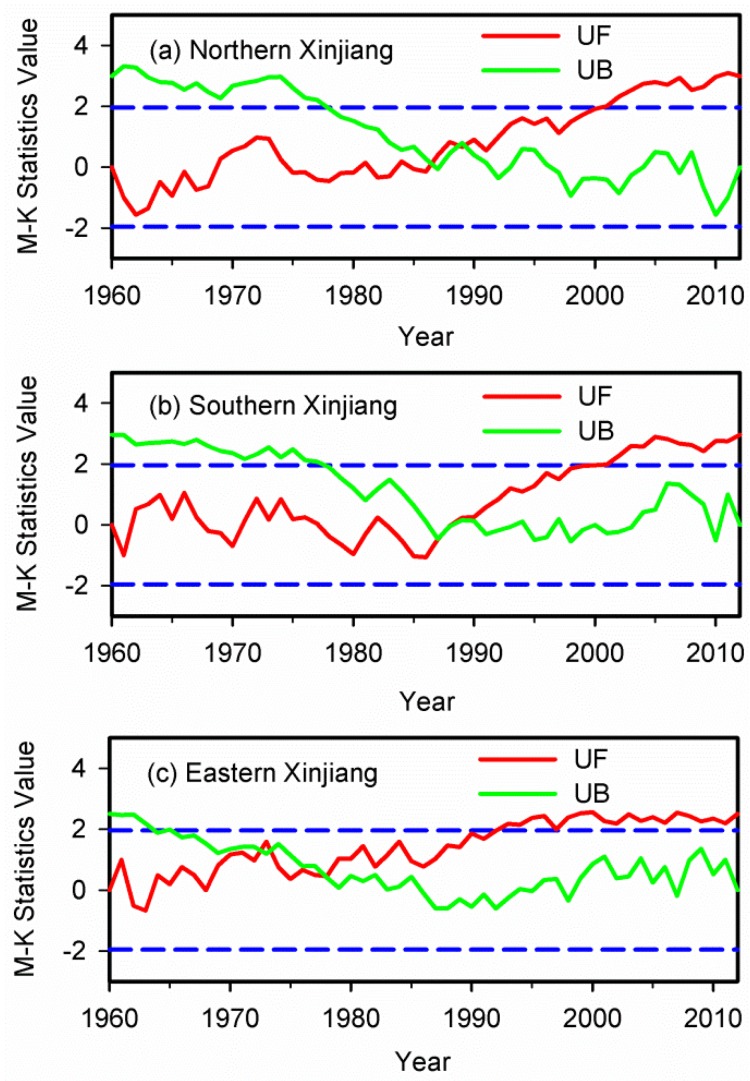
The M-K analysis results of average annual precipitation at 95% confidence level (the blue dash dotted lines) in (**a**) northern; (**b**) southern and (**c**) eastern Xinjiang during 1960–2012.

We also analyzed the abrupt turning points of annual precipitation in northern, southern and eastern Xinjiang during 1960–2012. Figure 12 shows the M-K analysis results of the annual precipitation in these sub-regions at 95% confidence level. The test for annual precipitation in northern and southern Xinjiang shows that the abrupt turning points in annual precipitation occurred in 1987 in these regions. Two abrupt turning points were detected for annual precipitation in eastern Xinjiang, and the abrupt increasing step change was in 1973 and 1978, respectively.

## 4. Conclusions

We analyzed the multi-scale characteristics of regional precipitation change in Xinjiang during 1960–2012 using the EEMD method. Changes in regional precipitation in Xinjiang clearly presented an inter-annual scale (2- and 6-year quasi-periodic fluctuations) and inter-decadal scale (12- and 23-year quasi-periodic fluctuations) during 1960–2012. Obvious non-linear upward trends in regional precipitation in the north, south, east and the entire Xinjiang were identified and obtained from the trend component from EEMD. Our results demonstrated the robustness to reconstruct the inter-annual variation trend in regional precipitation in Xinjiang. A significance test was also performed to determine inherently different time scales of IMF components. Moreover, we calculated the variance contribution rate of each IMF and trend component for the precipitation anomaly which suggested that 2–3-year quasi-periodic fluctuation was dominant in regional precipitation and the inter-annual variation had a considerable effect on the overall precipitation variation in Xinjiang. We also found that there were distinctive spatial differences in variation trends and turning points of precipitation in Xinjiang. Compared to traditional decomposition methods, the EEMD method, without using any *a priori* determined basis functions, can effectively extract the reliable multi-scale fluctuations and reveal the intrinsic oscillation properties of climate elements.

However, there are still several limitations of the present study. Firstly, our study only presented some potential explanations for the multi-scale oscillations of precipitation without exploring the deeper mechanisms. Secondly, there were no convincing reasons for the spatial differences in variation trends and turning points of precipitation. Thirdly, contemporary soft computing techniques in water resources engineering such as artificial neural network [60,61,62,63,64] were not coupled with EEMD in our study. A future work should, therefore, explore the deeper mechanisms of multi-scale oscillations and explain the reasons for the spatial differences in variation trends and turning points of precipitation. Importantly, future efforts can focus on the development of a hybrid model by incorporating EEMD for hydrological time series forecasting [65,66,67], which will be helpful for effective water resources management.

## Figures and Tables

**Table 1:** Information of the meteorological stations used in this study.

Region	Station ID	Station Name	Longitude (°E)	Latitude (°N)	Altitude (m)
Northern Xinjiang	51053	Habahe (HBH)	86.40	48.05	532.6
	51059	Jmunai (JMN)	85.87	47.43	984.1
	51068	Fuhai (FH)	87.47	47.12	500.9
	51076	Altai (ALT)	88.08	47.73	735.3
	51087	Fuyun (FY)	89.52	46.98	807.5
	51133	Tacheng (TC)	83.00	46.73	534.9
	51156	Hebuksair (HBKS)	85.72	46.78	1291.6
	51186	Qinghe (QH)	90.38	46.67	1218.2
	51232	Alashankou (ALSK)	82.57	45.18	336.1
	51241	Tuoli (TL)	83.60	45.93	1077.8
	51243	Karamay (KRMY)	84.85	45.62	449.5
	51288	Beitashan (BTS)	90.53	45.37	1653.7
	51330	Wenquan (WQ)	81.02	44.97	1357.8
	51334	Jinghe (JH)	82.90	44.62	320.1
	51346	Wusu (WS)	84.67	44.43	478.7
	51356	Shihezi (SHZ)	86.05	44.32	442.9
	51365	Caijiahu (CJH)	87.53	44.20	440.5
	51379	Qitai (QT)	89.57	44.02	793.5
	51431	Yining (YN)	81.33	43.95	662.5
	51437	Zhaosu (ZS)	81.13	43.15	1851.0
	51463	Urumqi (UMQ)	87.65	43.78	935.0
	51477	Dabancheng (DBC)	88.32	43.35	1103.5
Southern Xinjiang	51467	Baluntai (BLT)	86.30	42.73	1739.0
	51542	Bayinbluk (BYBL)	84.15	43.03	2458.0
	51567	Yanqi (YQ)	86.57	42.08	1055.3
	51628	Aksu (AKS)	80.23	41.17	1103.8
	51633	Baicheng (BCH)	81.90	41.78	1229.2
	51642	Luntai (LT)	84.25	41.78	976.1
	51644	Kucha (KC)	82.97	41.72	1081.9
	51656	Korla (KL)	86.13	41.75	931.5
	51701	Turgat (TG)	75.40	40.52	3504.4
	51705	Wuqia (WQ)	75.25	39.72	2175.7
	51709	Kashi (KS)	75.98	39.47	1289.4
	51711	Ahqi (AHQ)	78.45	40.93	1984.9
	51716	Bachu (BC)	78.57	39.80	1116.5
	51720	Keping (KP)	79.05	40.50	1161.8
	51730	Alar (AL)	81.27	40.55	1012.2
	51765	Tieganlik (TGLK)	87.70	40.63	846.0
	51777	Ruoqiang (RQ)	88.17	39.03	887.7
	51804	Tashikurgan (TSKG)	75.23	37.77	3090.1
	51811	Shache (SC)	77.27	38.43	1231.2
	51818	Pishan (PS)	78.28	37.62	1375.4
	51828	Hotan (HT)	79.93	37.13	1375.0
	51839	Minfeng (MF)	82.72	37.07	1409.5
	51855	Qiemo (QM)	85.55	38.15	1247.2
	51931	Yutian (YT)	81.65	36.85	1422.0
Eastern Xinjiang	51495	Shisanjianfang (SSJF)	91.73	43.22	721.4
	51526	Kumishi (KMS)	88.22	42.23	922.4
	51573	Turpan (TP)	89.20	42.93	34.5
	52101	Balikun (BLK)	93.05	43.60	1677.2
	52118	Yiwu (YW)	94.70	43.27	1728.6
	52203	Hami (HM)	93.52	42.82	737.2
	52313	Hongliuhe (HLH)	94.67	41.53	1573.8

**Table 2:** Each IMF cycle and its variance contribution rate for precipitation in Xinjiang.

IMFs and Residue	IMF1	IMF2	IMF3	IMF4	RES
Period (year)	2	6	12	23	
Contribution Rate (%)	47.00	12.59	5.15	3.13	32.13

**Table 3:** The periods and variance contribution rate of each IMF for precipitation in different regions of Xinjiang.

Region	IMFs and Residue	IMF1	IMF2	IMF3	IMF4	RES
Northern Xinjiang	Period (year)	2 *	7	14	25	*
	Contribution Rate (%)	35.09	10.71	6.02	1.05	47.13
Southern Xinjiang	Period (year)	2 *	5	10	23	*
	Contribution Rate (%)	40.47	11.37	0.89	1.81	45.46
Eastern Xinjiang	Period (year)	2 *	6	12	21	*
	Contribution Rate (%)	47.93	7.69	1.38	1.74	41.26

* means significant at the 90% confidence level.
